# Separation Options for Phosphorylated Osteopontin from Transgenic Microalgae *Chlamydomonas reinhardtii*

**DOI:** 10.3390/ijms19020585

**Published:** 2018-02-16

**Authors:** Ayswarya Ravi, Shengchun Guo, Beth Rasala, Miller Tran, Stephen Mayfield, Zivko L. Nikolov

**Affiliations:** 1 Department of Biological and Agricultural Engineering, Texas A&M University, College Station, TX 77843, USA; raviays.10h@tamu.edu (A.R.); guoshengchun121@tamu.edu (S.G.); 2 Triton Algae Innovations, San Diego, CA 92121, USA; beth@tritonai.com (B.R.); miller@tritonai.com (M.T.); 3 California Center of Algae Biotechnology, University of California San Diego, San Diego, CA 92093, USA; smayfield@ucsd.edu

**Keywords:** recombinant osteopontin, *C. reinhardtii*, protein, phosphorylation, ceramic hydroxyapatite, gallium, Ga-IMAC, purification, OPN

## Abstract

Correct folding and post-translational modifications are vital for therapeutic proteins to elicit their biological functions. Osteopontin (OPN), a bone regenerative protein present in a range of mammalian cells, is an acidic phosphoprotein with multiple potential phosphorylation sites. In this study, the ability of unicellular microalgae, *Chlamydomonas reinhardtii*, to produce phosphorylated recombinant OPN in its chloroplast is investigated. This study further explores the impact of phosphorylation and expression from a “plant-like” algae on separation of OPN. Chromatography resins ceramic hydroxyapatite (CHT) and Gallium-immobilized metal affinity chromatography (Ga-IMAC) were assessed for their binding specificity to phosphoproteins. Non-phosphorylated recombinant OPN expressed in *E. coli* was used to compare the specificity of interaction of the resins to phosphorylated OPN. We observed that CHT binds OPN by multimodal interactions and was better able to distinguish phosphorylated proteins in the presence of 250 mM NaCl. Ga-IMAC interaction with OPN was not selective to phosphorylation, irrespective of salt, as the resin bound OPN from both algal and bacterial sources. Anion exchange chromatography proved an efficient capture method to partially separate major phosphorylated host cell protein impurities such as Rubisco from OPN.

## 1. Introduction

The work horse of complex protein production for nearly 56% of recombinant proteins is the mammalian Chinese hamster ovary (CHO) cell line [1]. The ability of mammalian cells to properly fold and perform post-translational modifications (PTMs) of proteins, out-weighs the high cultivation costs and potential viral contamination issues. *Escherichia coli* is also extensively employed to produce recombinant proteins and does so at a fraction of the CHO cost but the resultant proteins often lack proper folding, PTMs and are prone to endotoxin contamination [2]. To overcome the disadvantages of mammalian and bacterial expression, the unicellular microalga *Chlamydomonas reinhardtii* has been explored as an alternative protein production platform. Specifically, the chloroplast of *C. reinhardtii* has been utilized for the production of difficult-to-express proteins that require specific folding and PTMs to exhibit biological function [3,4,5,6,7]. 

One such mammalian protein is osteopontin (OPN), also called bone sialoprotein-1 (BSP1), which is present in milk, bodily fluids and is responsible for bone development and regeneration. Other crucial functions of OPN include prevention of renal calcifications, protecting cells from apoptosis [8] and cytokine activity that differentially regulates inflammatory and immune response to allergic reactions [9]. The native mammalian form of OPN is an acidic, glycosylated sialoprotein, rich in aspartic acid, glutamic acid and serine residues [10]. Nascent bovine OPN is a ~34 kDa (~300 amino acid) protein, with an isoelectric point (pI) of 4.3, an arginine-glycine-aspartic acid (RGD) motif, a thrombin cleavage site, a polyaspartate domain and multiple calcium binding sites. In addition, twenty-eight potential phosphorylation sites and 3 glycosylation sites have been identified in the structure of OPN [11,12]. OPN’s phosphorylation apparently provides a stabilizing effect on the bond between calcium and hydroxyapatite in bones [13], regulates the interaction of OPN with integrins during signaling in CD44 mediated immune responses [14] and is vital for inhibiting vascular calcification in smooth muscle cells [15]. Therefore, to take advantage of the diverse beneficial activities of OPN, a potential recombinant protein production system should also be able to deliver optimal in vivo OPN phosphorylation.

Despite the immense potential of OPN in the healthcare industry, reports dealing with recovery, purification and commercial applications have been relatively few. Attempts at purifying OPN from tissues and bones have been confined to affinity-tag chromatography [16,17,18,19], which only allows the production of limited amounts for OPN characterization and functionality assays. 

Most of the methods employed to purify larger quantities of OPN, relied on generic protein properties such as hydrophobicity and ionic charge. Early reports with human milk employ weak anion exchange chromatography followed by several low capacity processing steps that resulted in low OPN yields due to losses from the multiple purification steps [20,21]. Currently, the primary source of OPN is bovine milk whey, which is processed by two consecutive anion exchange chromatography steps or anion exchange chromatography followed by two consecutive hydrophobic interaction chromatography steps [22,23]. Understandably, anion exchange adsorption is the default choice for capture and concentration of acidic OPN. It appears that consideration of other resin chemistries to increase process selectivity and yield warrants further investigation. The question to ask is whether the polyaspartate cluster (88–93 AA) and multiple phosphorylation sites in OPN can be exploited to achieve a more specific interaction with chromatographic adsorbents carrying transition metals and Ca^2+^ ions.

Therefore, the specific objectives of this study were (1) to assess the feasibility of chloroplast of the microalgae *C. reinhardtii* to produce phosphorylated OPN and (2) to evaluate OPN recovery and adsorption chromatography options that could take advantage of phosphorylation and/or polyaspartate sequences in the OPN structure. To evaluate the performance of algal-produced OPN, *E. coli* transformed with same OPN gene construct was used as benchmark.

## 2. Results and Discussion

### 2.1. Expression and Characterization of Recombinant Osteopontin in C. reinhardtii and E. coli

**Figure 1:**
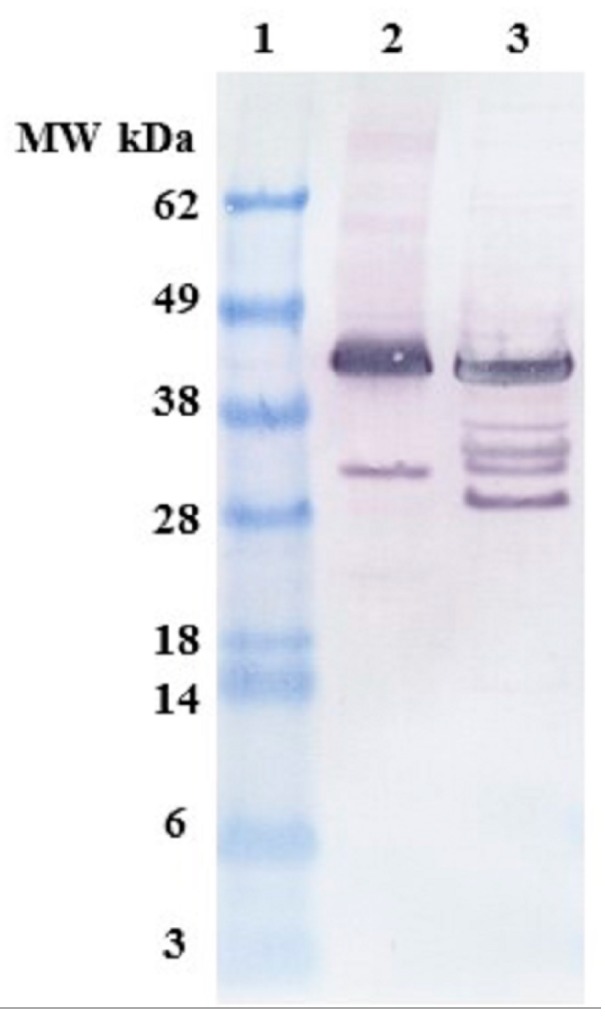
Anti-FLAG western blots showing *N*-terminal fragments of osteopontin (OPN). **Lane 1**. Molecular weight (MW) marker, **lane 2**. *C. reinhardtii* lysate diluted 4×, **lane 3**. *E. coli* lysate diluted 16×.

The transgenic cells of *C. reinhardtii* and *E. coli* expressing OPN were created by Mayfield’s group at California Center of Algae Biotechnology, University of California San Diego (UCSD) [24] and biomass was grown at Texas A&M University. Quantification of OPN concentration in *C. reinhardtii* lysates was difficult because of the low expression levels and interference of lysate components with enzyme-linked immunosorbent assay (ELISA). In the absence of a reliable quantification method, we relied on band density analysis of western blots with ImageJ software (v.1.49, U. S. National Institutes of Health, Bethesda, MD, USA), using FLAG-affinity purified OPN as a standard. *C. reinhardtii* OPN expression levels varied between biomass batches and was on average 4× lower than OPN in *E. coli* cells, as indicated by similar band densities of 4× and 16× diluted *C. reinhardtii* and *E. coli* cell lysates, respectively (Figure 1, lanes 2 & 3). In terms of initial OPN concentration expressed as a percent of total soluble protein (TSP), *E. coli* lysates contained 2.9% and algal lysates between 0.1% and 0.2% OPN.

Full-length recombinant OPN migrated to an apparent molecular weight of 45 kDa. This observation was consistent with the varied molecular weight (75–44 kDa) of OPN reported in literature and was attributed to the highly acidic nature of the protein [25,26]. OPN expressed in the chloroplast of *C. reinhardtii* (Lane 2) consists of a major 45 kDa band and an *N*-terminal 35 kDa fragment. In addition to the 45 kDa band, *E. coli* lysate (Lane 3) contains several *N*-terminal fragments with apparent molecular weights corresponding to 40, 38, 35, 30 and 28 kDa. There were considerably fewer *N*-terminal fragments observed in algal lysates as compared to *E. coli*, suggesting that OPN is better protected from fragmentation in the chloroplast of *C. reinhardtii* than in the *E. coli* cytosol.

**Figure 2:**
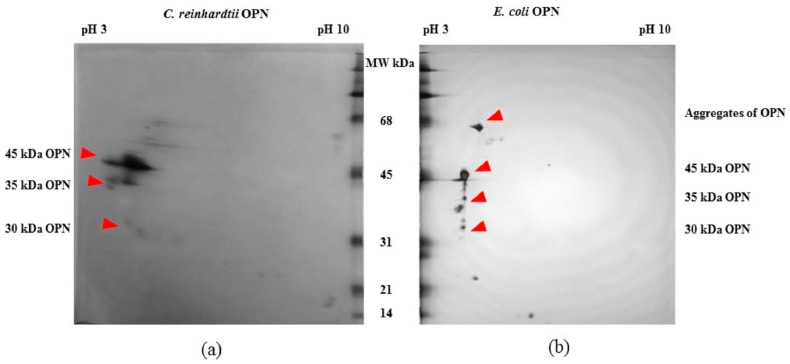
2D gel electrophoresis of OPN from transgenic (**a**) *C. reinhardtii* and (**b**) *E. coli* lysates obtained by FLAG-affinity purification.

The FLAG-affinity-purified *E. coli* and *C. reinhardtii* OPNs were analyzed by 2D electrophoresis gel (Figure 2) to estimate pI of various OPN forms. The 45 kDa OPN from *C. reinhardtii* resolved in a pI range from pH 3.5 to pH 4.5. A similar streak of varying pI (pH ~3.5 to 4.0) was observed with the 35 kDa fragment. The observed streaks suggest the presence of OPN isoforms that are likely due to heterogeneous post-translational phosphorylation and/or in vitro enzymatic de-phosphorylation. In contrast, *E. coli* 45 kDa OPN resolved into a spot localized at pH 4.5. The *E. coli* OPN fragments were also localized at approximately the same pH 4.5. Single spots instead of streaks of *E. coli* OPN is consistent with the expected lack of protein phosphorylation in *E. coli* [27,28,29]. Additional spots probably belonging to *E. coli* host protein were also detected on 2D gels.

**Figure 3:**
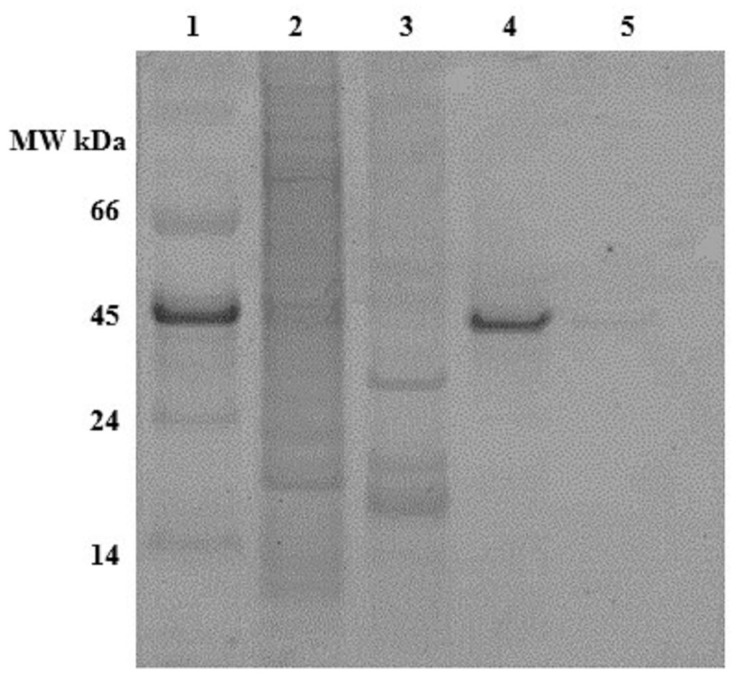
Pro-Q diamond phosphostained gel of OPN. **Lane 1**. MW marker, **lane 2**. *C. reinhardtii* lysate, **lane 3**. *E. coli* lysate, **lane 4**. Recombinant OPN purified from *C. reinhardtii*, **lane 5**. Recombinant OPN purified from *E. coli*. Each lane contains the same amount (~50 µg) of total soluble protein (TSP).

The presence or absence of phosphorylation on the recombinant OPN was detected by phosphostaining an SDS-PAGE gel (Figure 3). The stained band in lane 4 indicates that the FLAG-affinity purified 45 kDa OPN from *C. reinhardtii* is phosphorylated, while its counterpart from *E. coli* in lane 5 is not stained and, thus, not detectable. The phosphostained band in lane 1 belongs to 45 kDa ovalbumin, which served as a phosphorylated protein marker. The absence of phosphostaining of the 35 kDa band may indicate little or no phosphorylation.

The detection of phosphorylated residues in algal 45 kDa OPN provided motivation to look into the specificity of algal OPN interaction with ceramic hydroxyapatite (CHT) and immobilized transition metal (Ga, Fe, Zn) resins.

### 2.2. Ceramic Hydroxyapatite Chromatography

**Figure 4:**
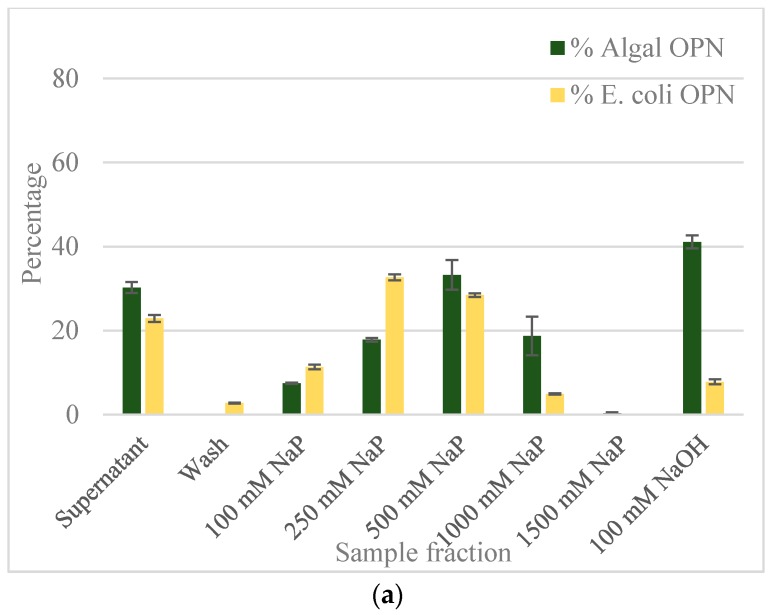

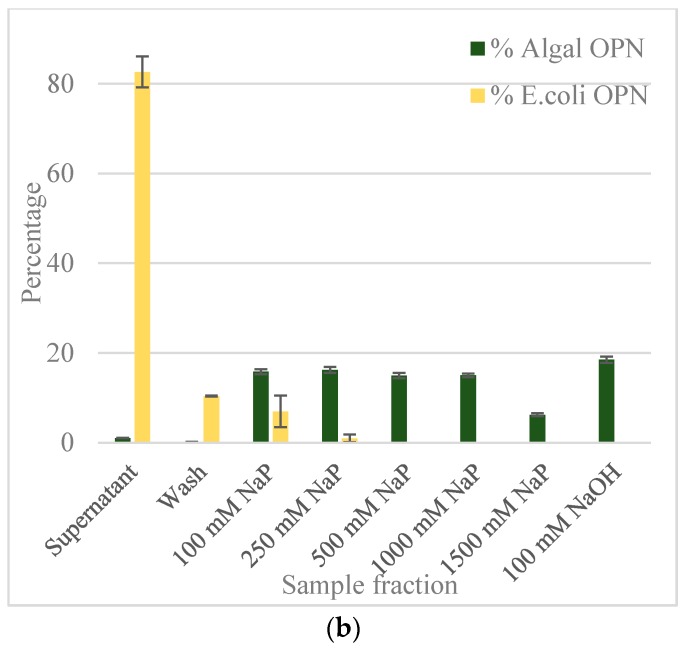
OPN distribution in fractions obtained with ceramic hydroxyapatite (CHT) resin (**a**) with no salt; (**b**) with 250 mM NaCl. Step elutions were performed with sodium phosphate (NaP) buffer, pH 6.8. Data (% OPN) were generated by ImageJ analysis of anti-FLAG western blots and normalized by the amount in the lysate. Error bars indicate standard deviation of triplicate ImageJ measurements.

The results from equilibrium adsorption of OPN to CHT resin from clarified *C. reinhardtii* and *E. coli* lysates are summarized in Figure 4.

**Figure 5:**
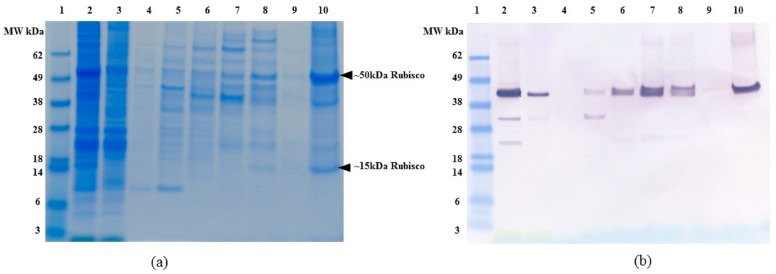
(**a**) SDS-PAGE and (**b**) Anti-FLAG western blot of *C. reinhardtii* lysate binding and elution profile from ceramic hydroxyapatite (CHT) resin without 250 mM NaCl. All samples diluted to <1 mg/mL TSP. **Lane 1**: MW marker, **lane 2**. Clarified lysate, **lane 3**. Supernatant, **lane 4**. Washes 3 column volumes (CV), **lane 5**. Elution with 100 mM NaP, **lane 6**. Elution with 250 mM NaP, **lane 7**. Elution with 500 mM NaP, **lane 8**. Elution with 1000 mM NaP, **lane 9**. Elution with 1500 mM NaP, **lane 10**. Elution with 100 mM NaOH. All elutions were performed with 5 CV of the respective buffer.

The adsorbed *E. coli* and *C. reinhardtii* OPN, respectively, were eluted using increasing strength (100 to 1500 mM) of NaP buffer. The bar graph in Figure 4a reveals that the majority (>70%) of OPN from both lysates was adsorbed to the CHT resin in the absence of salt. The relatively low amount of *C. reinhardtii* OPN detected in the supernatant (30%) and its absence in the wash fractions indicates strong interactions between OPN and CHT. The bound *C. reinhardtii* OPN began to elute with 100 mM NaP and continued eluting through 1000 mM phosphate concentration. A significant fraction of adsorbed OPN eluted with the 250 mM (18%) and 500 mM NaP (33%) steps, respectively. Since we could not close the mass balance on OPN in the eluted fractions, even after 5 column volumes (CV) elution with 1500 mM NaP, we resorted to harsher desorption conditions using 100 mM NaOH. The strongly adsorbed OPN, estimated at 41% of initial OPN by ImageJ analysis, was completely eluted with 100 mM NaOH. Western blots (Figure 5b) show that the 35 kDa fraction was eluted with 100 mM NaP and that the 45 kDa form interacted even more strongly, requiring higher phosphate concertation >250 mM NaP and 100 mM NaOH for desorption. 

As the bar graph in Figure 4a indicates, about 23% of *E. coli* OPN remained in the supernatant and 3% was found in the wash fractions. In contrast to *C. reinhardtii*, 72% of *E. coli* OPN eluted from hydroxyapatite resin in the 100–500 mM phosphate buffer range. As expected from the mass balance of OPN fractions eluted with the three NaP steps changes, the application of 100 mM NaOH did not yield a significant amount of OPN (only 8% of initial amount). The significant difference in the amount of 45 kDa OPN eluted with 100 mM NaP (7% algal OPN consisting of mainly 35 kDa OPN vs. 11% of *E. coli* 45 kDa OPN) and 100 mM NaOH (41% algal OPN vs. 8% *E. coli* OPN) steps indicate that *E. coli* and *C. reinhardtii* OPN interact differently with heterogeneous binding sites of CHT resin.

To determine if the different interaction strengths of OPN with CHT were due to phosphorylation i.e. phosphorylated (*C. reinhardtii*) vs. non-phosphorylated (*E. coli*) OPN, the adsorption and elution experiments were repeated in the presence of 250 mM NaCl (Supplementary data Figure S1). The inclusion of 250 mM NaCl in the adsorption and elution buffers was intended to reduce ionic interactions of amino acid side chains of OPN with Ca^2+^ and PO_4_^3−^ sites on the CHT without disrupting the much stronger coordinate bonding between OPN’s phosphoryl groups and Ca^2+^ on CHT surface. The results shown in Figure 4b clearly demonstrate a difference in the adsorption of *E. coli* and *C. reinhardtii* OPN in the presence of salt. About 83% of *E. coli* OPN remains in the supernatant at the end of the adsorption period compared to the barely detectable amount (~1%) OPN in *C. reinhardtii* supernatants. An additional 10% of *E. coli* OPN was removed in the wash step while none of *C. reinhardtii* OPN was desorbed during the washing. Comparison of the data presented in Figure 4 demonstrates that the adsorption of *E. coli* OPN to CHT was significantly weakened by 250 mM NaCl, whereas that of *C. reinhardtii* was not. The latter, somewhat atypical behavior of increased *C. reinhardtii* OPN binding, can be explained by NaCl suppression of electrostatic repulsions between phosphoryl groups on OPN and PO_4_^3−^ sites on the CHT [30]. The suppression of electrostatic repulsions, results in enhanced interactions between algal OPN’s phosphoryl groups with the Ca^2+^ on CHT surface. 

**Figure 6:**
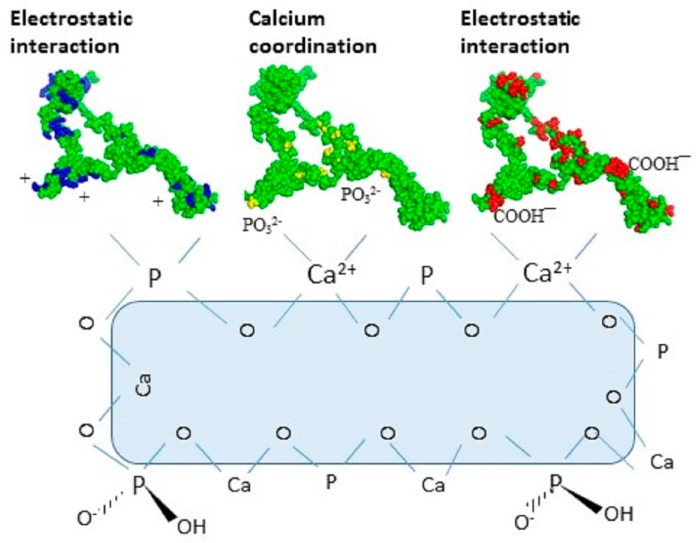
Proposed multimodal interaction mechanisms of OPN with ceramic hydroxyapatite (CHT) resin at pH 6.8. Basic (arginine, histidine and lysine) amino acids of OPN are shown in blue, phosphoryl groups in yellow and acidic (aspartic and glutamic acid) in red.

Therefore, *E. coli* OPN probably binds to hydroxyapatite by electrostatic interactions as depicted in Figure 6 below. This observation is consistent with the reported multi-modal interaction of proteins with CHT via charge interactions between acidic and basic amino acid side chains with the Ca^2+^ and PO_4_^3−^ moieties on CHT, respectively, which can be reversed by increasing the ionic strength of the buffer [31].

Based on these data, we believe that protein phosphorylation made a difference in the interaction of OPN with ceramic hydroxyapatite resin for the following reasons. First, compared to *E. coli* OPN, the addition of NaCl had a minimal or no measurable effect on *C. reinhardtii* OPN adsorption and elution from CHT (Figure 4). The presence of NaCl weakens *E. coli* OPN interaction with CHT as evidenced by 83% of OPN remaining unbound in supernatant and the residual 17% being desorbed during wash and first step 100 mM NaP step (Figure 4b). Second, the elution of *C. reinhardtii* OPN, irrespective of ionic strength, required displacement by increasing phosphate concentration and complete desorption of phosphorylated OPN by the application of 100 mM NaOH. This line of evidence suggests that *C. reinhardtii* OPN adsorption to CHT is mediated by the stronger Ca^2+^–PO_3_^2−^ coordinate binding rather than simple charge interactions. And, the unusually strong adsorption of the 45 kDa *C. reinhardtii* OPN form, which required 100 mM NaOH for elution (Figure 5b), is probably the result of interaction of multiple, closely positioned phosphoryl (PO_3_^2−^) groups on OPN molecule with calcium moieties (Ca^2+^) of CHT resin.

To assess the specificity and purification capability of CHT, we compared host protein binding and elution by SDS-PAGE (Figure 5a). Data in Figure 5a indicate that a significant number of algal proteins would co-elute with OPN. The major OPN “competitor” appears to be acidic, phosphorylated chloroplast protein, Rubisco [32], as large (~50 kDa) and small subunit (~15 kDa) can be seen in 1000 mM NaP and 100 mM NaOH fractions (Figure 5a, lane 8 and 10 respectively). The identity of Rubisco was confirmed by western blots using anti-Rubisco large and small subunit antibodies (Supplementary data Figure S3). Thus, the presence of Rubisco in *C. reinhardtii* lysates would eventually reduce the resin breakthrough capacity and OPN purification fold, both of which are important factors in process scale-up. A possible solution to CHT application for OPN purification is to remove Rubisco and other interfering host protein impurities from the clarified lysates before OPN binding to CHT.

### 2.3. Gallium-Immobilized Metal Affinity Chromatography (Ga-IMAC)

**Figure 7:**
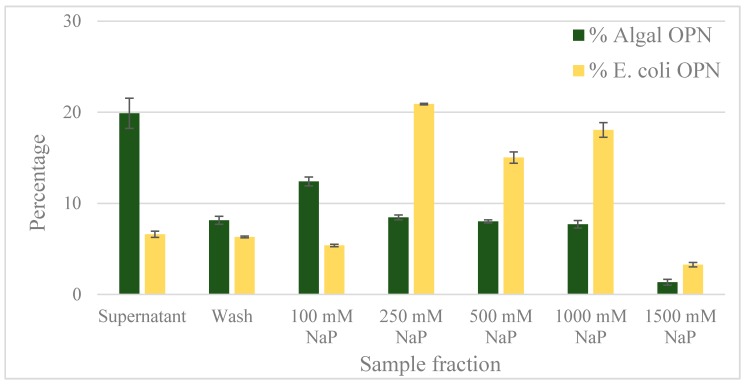
OPN distribution in fractions obtained with Ga-IMAC resin. Adsorption and elution was done in the presence of 500 mM NaCl. Step elutions were performed with sodium phosphate (NaP) buffer, pH 5.5. Data (% OPN) were generated by ImageJ analysis of anti-FLAG western blots and normalized by the amount in the lysate. Error bars indicate standard deviation of triplicate ImageJ measurements.

Gallium, iron and zinc immobilized on Chelating Sepharose are known to bind phospho-peptides by coordinate bonds [33,34,35]. Gallium, having higher affinity than other metals for phosphorylated peptides [36], has been selected in this study to determine if differential interactions between Ga^3+^ and phosphorylated protein residues could be utilized for the separation of *C. reinhardtii* OPN from non-phosphorylated proteins. As before, *E. coli* OPN has been used to assess the specificity of OPN–Ga interactions due to phosphorylation. To eliminate any ionic interactions between the protein and Ga-IMAC resin, OPN adsorption and elution was tested in the presence of 150 mM and 500 mM NaCl. Results from Ga-IMAC batch adsorption chromatography are shown in Figure 7. Approximately 20% of *C. reinhardtii* OPN remained unbound in the supernatant compared to only 7% of *E. coli* OPN. An additional 8% of algal OPN was removed during the resin wash step. The majority of the unbound and wash fractions contained 35 kDa form of algal OPN (Supplementary data Figure S2) which, as discussed above, did not bind strongly to CHT either. The adsorbed *C. reinhardtii* OPN started to elute with 100 mM NaP with 8%–12% of 45 kDa OPN continuing to elute in each step elution up to 1000 mM NaP concentration. The major fraction (21%) of bound *E. coli* OPN was in the second step elution with 250 mM NaP (Figure 7). The remaining bound *E. coli* OPN was eluted with 500 and 1000 mM NaP. 

The binding mechanism of *C. reinhardtii* OPN to Ga-IMAC apparently does not involve phosphorylated residues because non-phosphorylated *E. coli* OPN binds and elutes similarly to phosphorylated *C. reinhardtii* OPN (Figure 7). In addition, the similar binding and elution profiles observed with Fe-IMAC resin suggests that OPN interacts with Ga^3+^ and Fe^3+^ via the multiple carboxylic acidic containing amino acids occurring in close proximity (carboxyl clusters) present in both *E. coli* and C. *reinhardtii* OPN. Unfortunately, we could not confirm this hypothesis because adsorption at or below pH 4.5 (pKa of carboxyl groups) was not possible due to OPN precipitation at that pH range. Based on our data, it appears that protein interactions with transition metals (Ga^3+^ or Fe^3+^) are not confined specifically to phosphorylated residues and, therefore, the application of Ga-IMAC resin would not be useful for the separation of *C. reinhardtii* OPN from other acidic algae proteins present in the lysates.

### 2.4. Anion Exchange Chromatography

**Figure 8:**
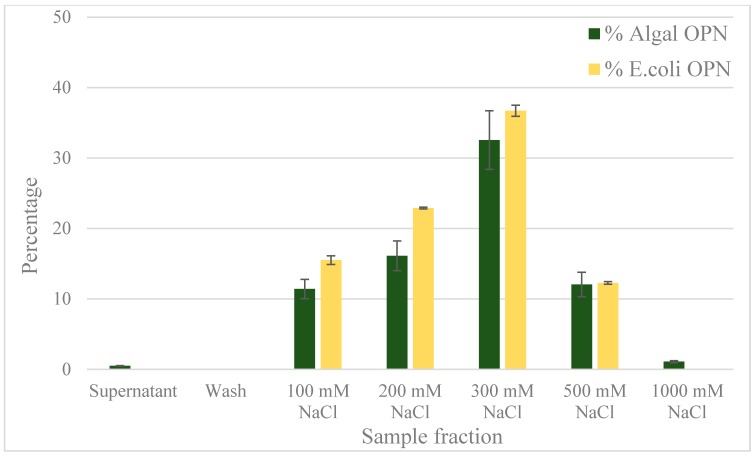
OPN distribution in fractions obtained with strong anion exchange resin (Capto Q). Step elutions were performed with sodium chloride in 50 mM Tris pH 7.0. Data (% OPN) were generated by ImageJ analysis of anti-FLAG western blots and normalized by the amount in the lysate. Error bars indicate standard deviation of triplicate ImageJ measurements.

**Figure 9:**
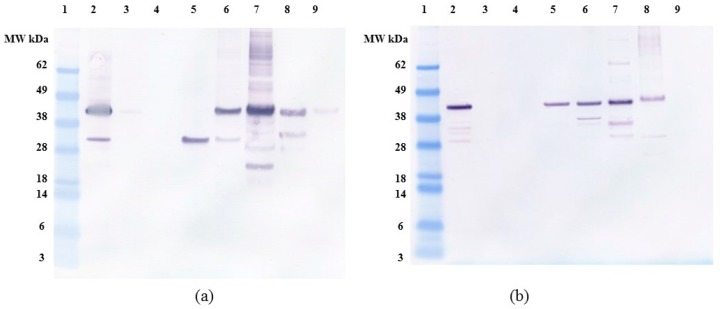
Anti-FLAG western blot of (**a**) *C. reinhardtii* (**b**) *E. coli* OPN binding and elution profile from Capto Q (strong anion exchange) resin. All samples diluted to <1 mg/mL TSP. **Lane 1**. MW marker, **lane 2**. Clarified lysate, **lane 3**. Supernatant, **lane 4**. Washes (3 CV), **lane 5**. Elution with 100 mM NaCl, **lane 6**. Elution with 200 mM NaCl, **lane 7**. Elution with 300 mM NaCl, **lane 8**. Elution with 500 mM NaCl, **lane 9**. Elution with 1000 mM NaCl. All elutions were performed with 5 CV of the respective buffer.

Previous studies with *E. coli* OPN have used anion exchange chromatography as one of the purification steps [37,38]. The use of anion exchange adsorption is a natural choice for highly acidic proteins like OPN. We decided to compare the interaction of *C. reinhardtii* and *E. coli* OPN with a strong anion exchange resin (Capto Q, GE Healthcare Life Sciences, Marlborough, MA, USA). The analysis of binding and elution pools of OPN from both sources are summarized in the bar graph (Figure 8) and western blots (Figure 9). When bound at pH 7.0 and a low conductivity of 4 mS, OPN bound strongly and specifically to the resin. No *C. reinhardtii* OPN (<1%) was detected by western blot in the supernatants and the washes (Figure 8). The majority of *C. reinhardtii* 35 kDa OPN fragment, which constitutes 11% total OPN was eluted with 100 mM NaCl (Figure 9a lane 5) suggesting weaker interaction with the quaternary amine than the intact 45 kDa form. The 45 kDa OPN from *C. reinhardtii* eluted in 200–500 mM NaCl range reaching a peak concentration in the 300 mM NaCl elution fraction. A faint band (~1.4%) was also observed with 1000 mM NaCl elution (Figure 9a lane 9). *E. coli* OPN also bound on anion exchange column strongly, demonstrated by the lack of a band in the supernatant and washes (Figure 9b lane 3 and 4). The elution of intact 45 kDa *E. coli* OPN starts earlier than algal OPN, with 16% in 100 mM NaCl elution (Figure 9b, lane 5) and elution continues till 500 mM NaCl with the highest concentration (37%) at 300 mM NaCl elution similar to *C. reinhardtii* OPN. The elution region for *E. coli* OPN is narrower under the same conditions with very little to no OPN over 500 mM NaCl elution. This observation corresponds to the 2D gel electrophoresis results, where *E. coli* OPN fragments were localized to spots, reflecting homogenous pI that should result in a uniform binding strength to the Capto Q resin. *C. reinhardtii* OPN had horizontal streaks on the 2D gel indicating a broader pI range of OPN due to heterogeneous phosphorylation. Hence, the elution profile of the adsorbed OPN would require a broader range of salt concentration to break the “heterogeneous” strength interactions with the resin. The ionic strength difference in the onset of 45 kDa OPN elution, 200 mM (*C. reinhardtii*) vs. 100 mM NaCl (*E. coli* OPN), probably reflects higher acidity of phosphorylated *C. reinhardtii* OPN. 

**Figure 10:**
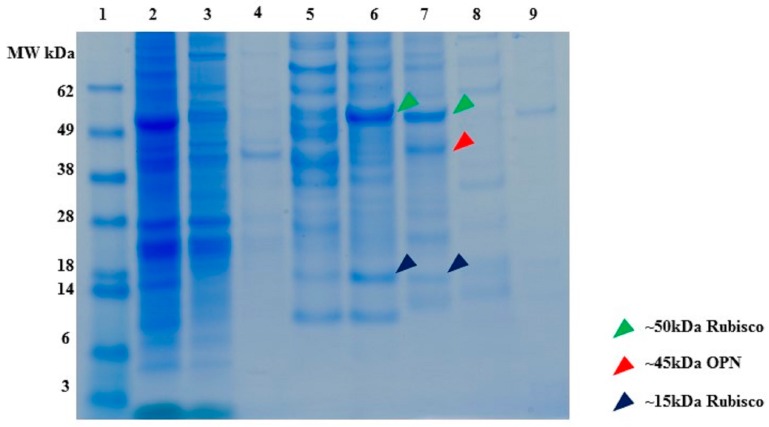
SDS-PAGE of *C. reinhardtii* lysate binding and elution profile from Capto Q (strong anion exchange) resin. All samples diluted to <1 mg/mL TSP. **Lane 1**. MW marker, **lane 2**. Clarified lysate, **lane 3**. Supernatant, **lane 4**. Washes (3 CV), **lane 5**. Elution with 100 mM NaCl, **lane 6**. Elution with 200 mM NaCl, **lane 7**. Elution with 300 mM NaCl, **lane 8**. Elution with 500 mM NaCl, **lane 9**. Elution with 1000 mM NaCl. All elutions were performed with 5 CV of the respective buffer.

SDS-PAGE (Figure 10) reveals that partial separation of OPN from *C. reinhardtii* host protein by anion exchange chromatography is possible. The majority (>99%) of recombinant algal OPN was adsorbed on the resin, about 49% of the host protein was present in the supernatant (lane 3) and wash fractions (lane 4) while the rest of the host protein was co-adsorbed with OPN. The majority of host protein was eluted in 100–300 mM NaCl fractions (lanes 5–7), whereas, OPN eluted in 200–500 mM NaCl fractions (lanes 6–8). Thus, by pooling 200 to 500 mM NaCl elution fractions at least forty-fold increase of OPN purity (from 0.2% to 8% TSP) and 80% recovery can be achieved by anion exchange chromatography. The anion exchange data also demonstrate that a substantial fraction of phosphorylated host protein (Rubisco), which was identified above as the major interfering host protein for OPN purification with CHT, can be partially separated from OPN by excluding the 200 mM NaCl elution and pooling only the 300 and 500 mM fraction for further OPN isolation. In the latter case, OPN purity would increase to 20% at the expense of reduced recovery (<60%) as calculated from Figure 10.

## 3. Materials and Methods

### 3.1. Cultivation of Recombinant Chlamydomonas reinhardtii Strain Expressing Bovine OPN

The chloroplast genome of *C. reinhardtii* strain cc1670 was modified to express bovine OPN by California Center of Algae Biotechnology, UCSD [24]. The *psbA* gene was replaced with cDNA for bovine OPN (GenBank accession number NP_776612.1) by homologous recombination. The modified strain was rescued by re-insertion of the *psbA* gene at a different location in the genome. A 1×-FLAG epitope tag (DYKDDDDKS) was inserted at the *N*-terminal of OPN sequence for identification and quantification, with an enterokinase cleavage site to facilitate removal after OPN purification.

Recombinant algal cells were grown on 15% Tris-Acetate-Phosphate (TAP) agar plates at room temperature. The colonies were transferred into TAP media containing 0.1% Hutner’s trace solution (*C. reinhardtii* research center) and 150 µg/mL ampicillin [39,40]. The cells were grown in sterile conditions with constant 125 rpm shaking, 200–300 µmols m^−2^·s^−1^ of cool white light at room temperature. They were harvested in mid-log phase at OD_750_ = 0.5 by centrifugation at 3500 rpm and 4 °C (Allegra 25R, Beckman Coulter, Brea, CA, USA). The wet biomass was stored at −80 °C.

### 3.2. Recombinant E. coli Strain and Cultivation

*E. coli* strain BL21 was modified to express bovine OPN with an *N*-terminal FLAG sequence for OPN identification and quantification similar to *C. reinhardtii* by California Center of Algae Biotechnology, UCSD. The recombinant strain was ampicillin resistant and OPN expression was controlled by the arabinose promotor. 

1 mL glycerol stock of the recombinant strain was used to grow overnight inoculum (5 mL) in Luria-Bertani (LB) media (150 µg/mL ampicillin) at 37 °C and 200 rpm. The overnight inoculum was transferred to 1 L Terrific Broth (Sigma-Aldrich, St. Louis, MO, USA, Cat # T0918) and *E. coli* cells were grown at 37 °C, 200 rpm to OD_600_ = 0.8. OPN expression was induced overnight with 0.2% (*w*/*v*) arabinose at 37 °C and 200 rpm. The wet biomass was harvested by centrifugation and stored at −80 °C.

### 3.3. Batch Adsorption Chromatography and Purification Studies 

Frozen biomass was thawed to room temperature and suspended in 1 g biomass:10 mL lysis buffer (50 mM Tris, 150 mM NaCl, 1 mM Ethylenediaminetetraacetic acid (EDTA), pH 7.4) containing protease and phosphatase inhibitor cocktail pills (Roche, Mannheim, Germany). Cells were lysed by sonication (15 min for *C. reinhardtii* and 5 min for *E. coli*) in 30 s on/off intervals at 4 °C using a sonicator (Sonifier 250, Branson, Danbury, CT, USA) at 30% output control and 30% duty cycle with a micro probe (1/8” micro tip A3-561 Branson, Danbury, CT, USA). Cell lysates were centrifuged (10,000× *g* for 20 min at 4 °C, Allegra 25R centrifuge, Beckman Coulter, Brea, CA, USA) and cell-free supernatants were filtered through 0.22 µm sterile polyethersulfone membrane syringe filter (30 mm diameter, Genesee Scientific, San Diego, CA, USA) and this was considered the clarified lysate for chromatography experiments. Protease and phosphatase inhibitor cocktail pills (Roche, Mannheim, Germany) in their recommended concentrations were added to all buffers used in the chromatography experiments. 

FLAG-affinity chromatography. FLAG-affinity purification was used to obtain highly purified recombinant OPN for analysis. 1 mL of anti-FLAG affinity gel (Sigma-Aldrich, St. Louis, MO, USA, Cat # A4596) was equilibrated in lysis buffer for 30 CV and mixed with 5 mL of *C. reinhardtii* clarified lysate or 1 mL of *E. coli* clarified lysate. Incubation of resin and lysate was carried out for 2 h at 4 °C with continuous end-over-end mixing in a Glas-Col rotor (Glas-Col LLC, Terre Haute, IN, USA) at ~33 rpm (40% speed control). The resin was separated from the clarified lysate by centrifuging at 6000× *g* for 5 min and the resin was loaded on a Bio Spin disposable chromatography column (Bio-Rad, Hercules, CA, USA, Cat # 732-6008). Non-specifically bound proteins were removed by washing with 10 CV of 50 mM Tris, 150 mM NaCl, 1 mM EDTA, pH 7.4. Proteins bound to resin were eluted using 5 CV of 100 mM Glycine, 400 mM NaCl, pH 3.5. The pH of the eluted proteins was immediately raised to 8.0 by adding 50 µL of 1 M Tris-HCl, to avoid protein denaturation at low pH.

Ceramic hydroxyapatite chromatography. Clarified lysates were dialyzed against 5 mM sodium phosphate (NaP) pH 6.8 for 2 h at 4 °C using snake-skin membrane (3.5 kDa molecular weight cut-off, Thermo Fisher Scientific, Waltham, MA, USA, Cat #68035). The clarified lysate (2.5 mL of *C. reinhardtii* clarified lysate, 0.66 mL of *E. coli* clarified lysate) was incubated for 1 h at room temperature by end-over-end mixing with 0.5 mL of ceramic hydroxyapatite resin (Bio-Rad, Hercules, CA, USA, Cat # 1584000) pre-equilibrated in 5 mM NaP pH 6.8 and resin was loaded on a chromatography column. The resin was washed with 10 CV of 5 mM NaP pH 6.8 followed by protein elution using a step-wise increasing concentration (100 mM, 250 mM, 500 mM, 1000 mM and 1500 mM) of NaP buffers at pH 6.8 with 5 CV of each buffer concentration.

Immobilized Metal Affinity Chromatography (IMAC). Gallium chloride at 400 mM concentration was immobilized on Chelating Sepharose FF (GE Healthcare Life Sciences, Marlborough, MA, USA, Cat # 17-0575-01) as per manufacturer instructions and equilibrated with 50 mM MES, 500 mM NaCl pH 5.5. The *C. reinhardtii* and *E. coli* lysates were dialyzed against 50 mM MES, 500 mM NaCl pH 5.5 for 2 h at 4 °C using snake-skin membrane (3.5 kDa molecular weight cut-off). The dialyzed sample (2.5 mL of *C. reinhardtii* clarified lysate, 0.66 mL of *E. coli* clarified lysate) was incubated with 0.5 mL of Ga-IMAC resin for 1 h at room temperature and loaded on a chromatography column. The resin was washed with 10 CV of 50 mM MES, 0.5 M NaCl pH 5.5, followed by protein elution using a step-wise increasing concentration (100 mM, 250 mM, 500 mM, 1000 mM and 1500 mM) of NaP buffers with 5 CV of each buffer concentration.

Anion-exchange chromatography. The clarified lysates were dialyzed against 50 mM Tris pH 7.0 for 2 h at 4 °C using snake-skin membrane (3.5 kDa molecular weight cut-off), to bring the conductivity to 4 mS. The dialyzed samples (2.5 mL of *C. reinhardtii* clarified lysate, 0.66 mL of *E. coli* clarified lysate) were incubated for 1 h at room temperature by end-over-end mixing with 0.5 mL of Capto Q resin (GE Healthcare Life Sciences, Marlborough, MA, USA, Cat # 17-5316-10) equilibrated in 50 mM Tris pH 7.0. The resin was separated by centrifuging at 6000× *g* for 5 min and loaded on a chromatography column. The resin was washed with 10 CV of 50 mM Tris pH 7.0, followed by protein elution using a step-wise increasing concentration (100 mM, 200 mM, 300 mM, 500 mM, 1000 mM) of NaCl in 50 mM Tris pH 7.0 with 5 CV of each buffer concentration.

### 3.4. Analytical Methods

All total protein measurements were calculated using Bradford assay (Coomassie plus Assay kit, Thermo Fisher Scientific, Waltham, MA, USA, Cat #23236) on a microtiter plate format with bovine serum albumin (BSA) as standard. The absorption was measured at 595 nm using VersaMax microplate reader (Molecular Devices, San Jose, CA, USA).

Proteins in sample were visualized and quantified using SDS-PAGE under reducing conditions. Denatured protein sample was loaded on NuPAGE Novex 4%–12% Bis-Tris pre-cast gradient gels (Thermo Fisher Scientific, Waltham, MA, USA, Cat # NP0335BOX). The proteins were separated by electrophoresis for 35 min at constant 200 V. All proteins were visualized by SimplyBlue SafeStain Coomassie G-250 stain (Thermo Fisher Scientific, Waltham, MA, USA, Cat # LC6065). ImageJ software (v.1.49, U. S. National Institutes of Health, Bethesda, MD, USA) was used to calculate purity of proteins in each sample lane.

Anti-FLAG western blot were prepared by transferring protein bands from SDS-PAGE gels onto a nitrocellulose membrane using iBLOT transfer kit (Thermo Fisher Scientific, Waltham, MA, USA, Cat # IB401002) for 7 min. The membrane was blocked with 2.5% non-fat milk in 0.05% TBS-T for 1 h and washed 3× with 0.05% TBS-T. The blocked membrane was then incubated with anti-FLAG M2 alkaline phosphatase conjugated antibody solution (Sigma-Aldrich, St. Louis, MO, USA, Cat # A9469) diluted 1:2000 in 0.05% TBS-T for 1 h at room temperature on a gel rocker. The antibody solution was washed 3× with 0.05% TBS-T and developed for 2 min using a solution of NBT/BCIP developer pills (Sigma-Aldrich, St. Louis, MO, USA, Cat # B5655) dissolved in 10 mL of distilled water. Standard curves with FLAG-affinity purified OPN were used to estimate OPN concentrations when necessary. ImageJ software (v.1.49, National Institutes of Health, Bethesda, Maryland, MD, USA) was used to calculate relative amounts (%) of OPN in the fractions generated during adsorption chromatography experiments, normalized by the amount of OPN in the lysate. The 45 kDa and fragments of OPN were all included in calculating the amount of OPN in each fraction. Rubisco subunits were confirmed using similar western blotting protocol. Anti-Rubisco large and small subunit polyclonal antibodies (Agrisera antibodies, Vännäs, Sweden) were used as primary antibodies (1:5000) and anti-rabbit-alkaline phosphatase (Sigma-Aldrich, St. Louis, MO, USA) as secondary antibody (1:5000).

Direct ELISA was used to determine OPN concentration in *E. coli* samples using FLAG-BAP (Sigma-Aldrich, St. Louis, MO, USA, Cat # P7582) as standards. Samples were diluted in phosphate buffer saline (PBS) and incubated overnight at 4 °C on Nunc immunosorbent 96-well plates. After 3 washes with PBS-T, blocking solution of 0.3% BSA in PBS was added to the 96-will plate and incubated for 2 h at 37 °C followed by similar wash and incubation with anti-FLAG M2 antibody conjugated with horse radish peroxidase (Sigma-Aldrich, St. Louis, MO, USA, Cat # A8592) diluted 1:5000 in PBS. TMB developer (Sigma-Aldrich, St. Louis, MO, USA, Cat # T0440) was used to develop the reaction and stopped with 2 M HCl, the absorbance was read at 450 nm. 

FLAG-affinity purified OPN samples at 50 µg/mL concentration were analyzed for their pI and molecular weight using 2D gel electrophoresis performed by Protein Chemistry Lab, Texas A&M University, USA. pI resolution was performed with immobilized pH gradient technology (IPG Dry Strip, GE Healthcare Life Sciences, Marlborough, MA, USA) followed by electrophoresis on GE Healthcare Tall Mighty Small system (8 × 10 cm). The resolved gel was silver stained to visualize OPN and specificity was confirmed by anti-FLAG western blot. The pI values of OPN were estimated using the standard curve established by the IPG Dry Strip manufacturer.

Phosphostaining was performed on FLAG-affinity purified OPN samples that were de-salted and de-lipidated by chloroform-methanol precipitation [41]. SDS-PAGE was performed as above and the gel was incubated in fixing solution (50% methanol, 10% acetic acid and 40% MilliQ water) overnight at room temperature on the gel rocker. The gel was washed 3× with MilliQ water and stained with Pro-Q diamond phosphostain (Thermo Fisher Scientific, Waltham, MA, USA, Cat # MPP33301) for 90 min in the dark. Destaining solution (20% acetonitrile in 50 mM sodium acetate pH 4.0) was added 3× with 30 min incubation each and the gel was finally washed 4× with MilliQ water. Gel imaging was performed on Typhoon Trio fluorescent imaging system (GE Healthcare Life Sciences, Marlborough, MA, USA) at excitation 532 nm green and 560 nm bandpass emission filter.

## 4. Conclusions

Osteopontin was successfully expressed in the chloroplast of *C. reinhardtii*, a novel alternative platform for recombinant protein production, capable of performing phosphorylation as a post-translational modification. The *C. reinhardtii* strain expressed two major OPN isoforms (45 kDa and 35 kDa) with pI ranging from 3.5 to 4.5 due to heterogeneous post-translational phosphorylation. 

Several chromatography resins were evaluated for isolating OPN by utilizing interactions with the phosphorylation and the unique polyaspartate sequence. Ceramic hydroxyapatite resin demonstrated affinity for acidic and phosphorylated proteins and adsorbed the phosphorylated OPN (45 kDa) isoform strongly. The adsorption and co-elution of phosphorylated host proteins like Rubisco along with *C. reinhardtii* OPN, reduced the purification efficiency of CHT resin. Since CHT could not distinguish between major phosphorylated impurities (Rubisco) and OPN, it is not ideal for separation of phosphorylated recombinant proteins from *C. reinhardtii*.

Gallium-IMAC, did not exhibit a preferential binding of phosphorylated OPN. Both phosphorylated OPN from *C. reinhardtii* and non-phosphorylated OPN from *E. coli* appeared to have similar binding and elution behavior with Ga-IMAC. Our study suggests that Ga^3+^ interacts with multiple carboxyl groups of OPN and host proteins and has no specific preference for the phosphorylated algal OPN 45 kDa form. As such, Ga-IMAC resin does not offer any advantage over CHT as a potential purification method for phosphorylated OPN forms. 

Anion exchange chromatography is a viable adsorption step for capturing OPN from both *E. coli* and *C. reinhardtii* clarified lysates. The anion exchange chromatography conducted under optimal adsorption, resin-wash and elution conditions removes as much as 50% of host protein while maintaining OPN recovery yield at 80%. Other anionic protein impurities bound to the resin can be partially separated from OPN based on their strength of charge interactions with the amine ligand resulting in a 20% pure OPN fraction.

Because of low accumulation levels in our current *C. reinhardtii* strain leading to low starting concentration of OPN in the clarified *C. reinhardtii* lysate (0.1%–0.2% TSP), the development of a scalable OPN purification train will probably require three or more chromatography steps. 

## Supplementary Materials

Supplementary materials can be found at http://www.mdpi.com/1422-0067/19/2/585/s1.

## Abbreviations

CHO	Chinese hamster ovary
PTM	Post-translational modification
OPN	Osteopontin
BSP	Bone sialoprotein
NaP	Sodium phosphate
CHT	Ceramic hydroxyapatite
Ga-IMAC	Gallium-immobilized metal affinity chromatography
Rubisco	Ribulose-1, 5-bisphosphate carboxylase/oxygenase
TSP	Total soluble protein
