# Supplementary material for: Separation Options for Phosphorylated Osteopontin from Transgenic Microalgae Chlamydomonas reinhardtii
Source: Int J Mol Sci. 2018 Feb 16;19(2):585. doi: 10.3390/ijms19020585 (PMC5855807; doi:10.3390/ijms19020585)
Supplement: Supplementary file 1 [file ijms-19-00585-s001.pdf]

Separation options for phosphorylated osteopontin from transgenic microalgae *Chlamydomonas reinhardtii*

Ayswarya Ravi [1], Shengchun Guo [1], Beth Rasala [2], Miller Tran [2], Stephen Mayfield [3] and Zivko L. Nikolov [1]*

Supplementary data

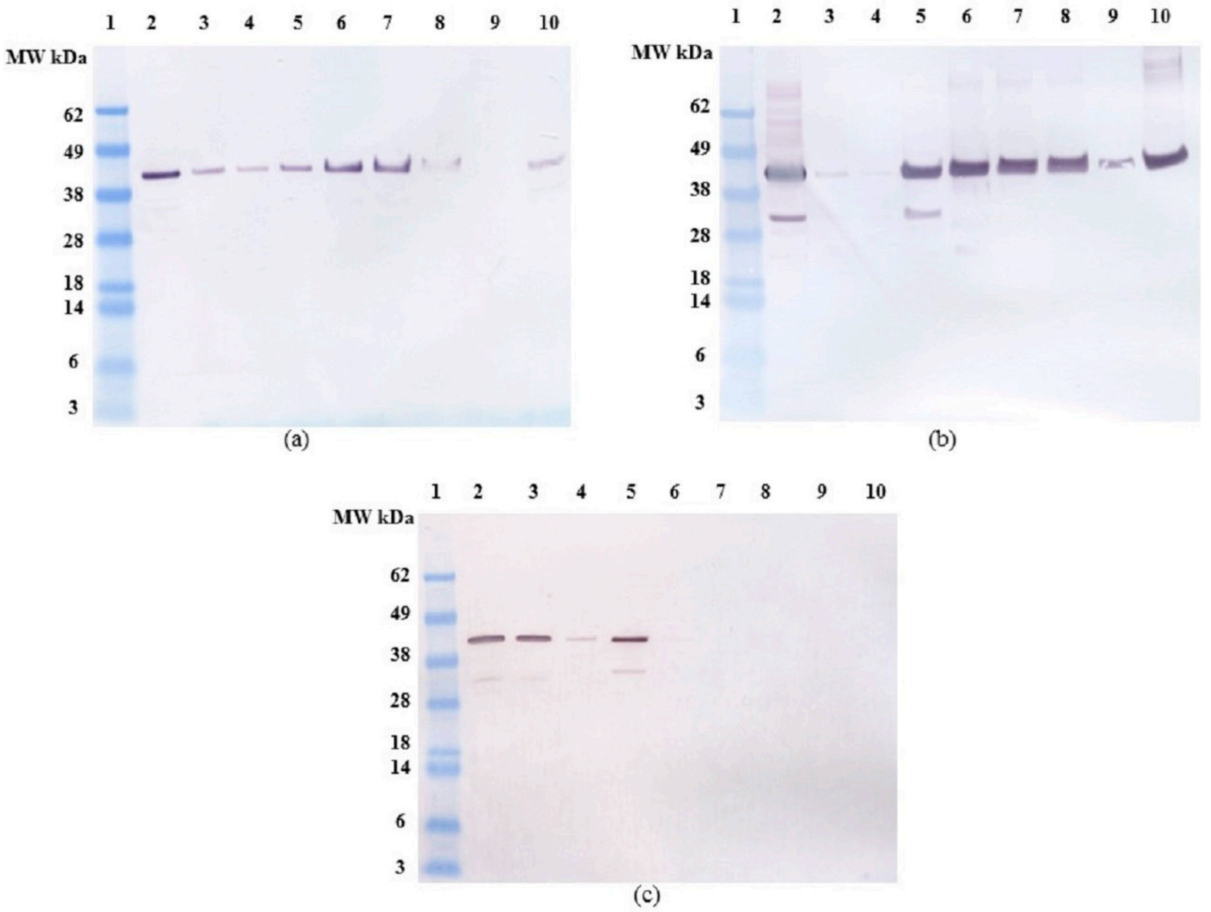

Figure S1. Anti-FLAG western blot of (a) *E. coli* OPN binding and elution profile without 250 mM NaCl (b) *C. reinhardtii* OPN binding and elution profile with 250 mM NaCl (c) *E. coli* OPN binding and elution profile with 250 mM NaCl from ceramic hydroxyapatite (CHT) resin. All samples diluted to <1 mg/mL total soluble protein (TSP). **Lane 1**. Molecular weight (MW) marker, **lane 2**. Clarified lysate, **lane 3**. Supernatant, **lane 4**. Washes 3 column volumes (CV), **lane 5**. Elution with 100 mM NaP, **lane 6**. Elution with 250 mM NaP, **lane 7**. Elution with 500 mM NaP, **lane 8**. Elution with 1000 mM NaP, **lane 9**. Elution with 1500 mM NaP, **lane 10**. Elution with 100 mM NaOH. All elutions were performed with 5 CV of the respective buffer.

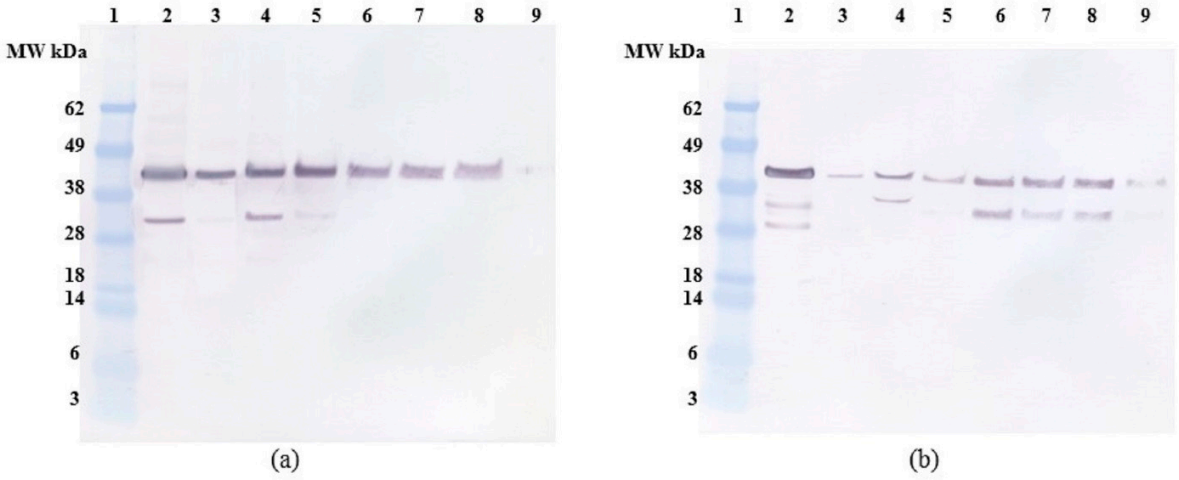

Figure S2. Anti-FLAG western blot of (a) *C. reinhardtii* OPN (b) *E. coli* OPN binding and elution profile from Ga-IMAC resin. All samples diluted to <1 mg/mL TSP. **Lane 1**. MW marker, **lane 2**. Clarified lysate, **lane 3**. Supernatant, **lane 4**. Washes (3 CV), **lane 5**. Elution with 100 mM NaP, **lane 6**. Elution with 250 mM NaP, **lane 7**. Elution with 500 mM NaP, **lane 8**. Elution with 1000 mM NaP, **lane 9**. Elution with 1500 mM NaP. All elutions were performed with 5 CV of the respective buffer.

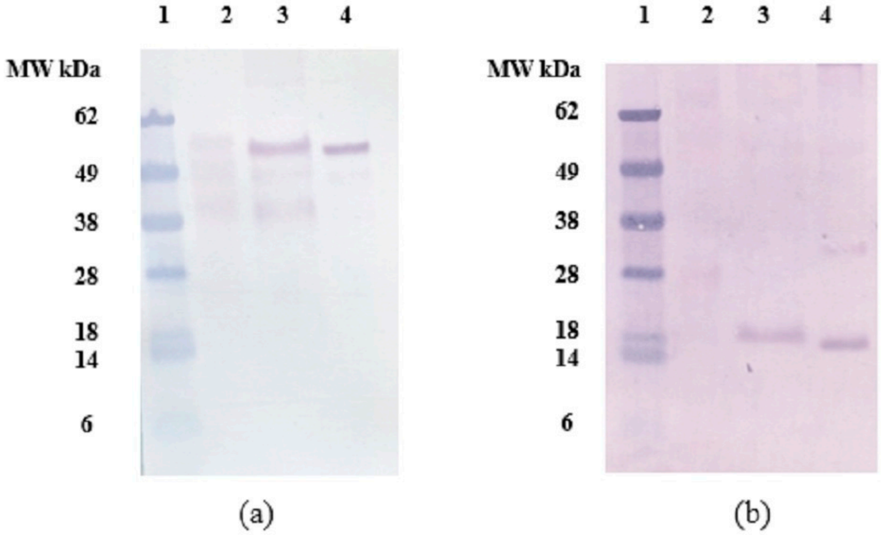

Figure S3. (a) Anti-Rubisco large subunit (b) Anti-Rubisco small subunit western blots of *C. reinhardtii* OPN samples. All samples diluted to <1 mg/mL TSP. **Lane 1**. MW marker, **lane 2**. *C. reinhardtii* clarified lysate, **lane 3**. Elution with 100 mM NaOH from ceramic hydroxyapatite (CHT) resin, **lane 4**. Elution with 200 mM NaCl from Capto Q resin. All elutions were performed with 5 CV of the respective buffer.
